# Selective FFR_CT_ testing in suspected stable angina in clinical practice - initial experiences

**DOI:** 10.1007/s10554-024-03214-8

**Published:** 2024-09-11

**Authors:** Shifan Thangavel, Kristian Taekker Madsen, Niels Peter Rønnow Sand, Karsten Tange Veien, Lone Deibjerg, Majed Husain, Susanne Hosbond, Dilek Hunerel Alan, Kristian Altern Øvrehus, Anders Junker, Jonas Mortensen, Kristian Korsgaard Thomsen, Lisette Okkels Jensen, Tina Svenstrup Poulsen, Flemming Hald Steffensen, Allan Rohold, Martin Busk

**Affiliations:** 1 Department of Cardiology, University Hospital of Southern Denmark, Vejle and Kolding, Denmark; 2 Department of Cardiology, University Hospital of Southern Denmark, Esbjerg, Denmark; 3 Department of Cardiology, Odense University Hospital, Odense, Denmark; 4 Institute of Regional Health Research, University of Southern Denmark, Odense, Denmark; 5 Department of Cardiology, University Hospital Aarhus, Aarhus Palle Juul-Jensens Blvd. 99, Aarhus, 8200 Denmark

**Keywords:** Angina pectoris, Myocardial fractional flow reserve, Computed tomography, Xray, Coronary angiography, Myocardial revascularization

## Abstract

Coronary CT angiography (CTA) derived fractional flow reserve (FFR_CT_) is recommended for physiological assessment in intermediate coronary stenosis for guiding referral to invasive coronary angiography (ICA). In this study, we report real-world data on the feasibility of implementing a CTA/FFR_CT_ test algorithm as a gatekeeper to ICA at referral hospitals. Retrospective all-comer study of patients with new onset stable symptoms and suspected coronary stenosis (30–89%) by CTA. Evaluation of CTA datasets, interpretation of FFR_CT_ analysis, and decisions on downstream testing were performed by skilled CT-cardiologists. CTA was performed in 3974 patients, of whom 381 (10%) were referred directly to ICA, whereas 463 (12%) to non-invasive functional testing: FFR_CT_ 375 (81%) and perfusion imaging 88 (19%). FFR_CT_ analysis was rejected in 8 (2%) due to inadequate CTA image quality. Number of patients deferred from ICA after FFR_CT_ was 267 (71%), while 100 (27%) were referred to ICA. Obstructive coronary artery disease (CAD) was confirmed in 62 (62%) patients and revascularization performed in 53 (53%). Revascularization rates, n (%), were higher in patients undergoing FFR_CT_-guided versus CTA-guided referral to ICA: 30–69% stenosis, 28 (44%) versus 8 (21%); 70–89% stenosis, 39 (69%) versus 25 (46%), respectively, both *p* < 0.05. Implementation of FFR_CT_ at referral hospitals was feasible, reduced the number of invasive procedures, and increased the revascularization rate.

## Introduction

In Denmark, as stated in international guidelines, coronary computed tomography angiography (CTA) has for years been used as a first-line test for evaluation of patients presenting with symptoms suggestive of new onset stable angina pectoris (SAP) [1]. CTA has proved superior to traditional non-invasive testing algorithms in reducing long-term incidence of myocardial infarction [2]. However, as CTA is a strict anatomic test and the correlation between stenosis severity and impact on coronary flow as measured by fractional flow reserve (FFR) is only moderate [3], additional non-invasive functional testing is recommended prior to referral to invasive assessment in stable patients with suspected coronary stenosis, unless stenosis severity and patient symptoms calls for direct invasive assessment [4, 5].

FFR derived from CTA (FFR_CT_) is a contemporary modality that allows physiological estimation of the impact on blood flow by coronary artery disease (CAD) detected by coronary CTA [6, 7]. This novel non-invasive modality has been validated for functional assessment of intermediate coronary stenosis [3, 8]. FFR_CT_ has demonstrated high and superior diagnostic performance compared to CTA alone [3], improved diagnostic sensitivity as compared to commonly applied stress perfusion imaging modalities [9–11], a high per-patient and -vessel agreement with invasive FFR [3, 8], and favourable prognostic outcomes in case of a normal FFR_CT_ test result [12–15]. Consequently, FFR_CT_ has recently been recommended for guiding referral to invasive coronary angiography (ICA) in patients with SAP and intermediate coronary stenosis by CTA [5, 16, 17]. However, only few hospitals have reported data on the applicability of FFR_CT_ consecutively in an all-comer setting. [14, 18, 19]. In this study of patients undergoing CTA at referral hospitals as a first-line test for suspected SAP, we evaluated the gate-keeping potential of using selective FFR_CT_ as the preferred second-line test in terms of feasibility, use of downstream procedures, and revascularization practice.

## Methods

### Study design and patient population

**Fig. 1:**
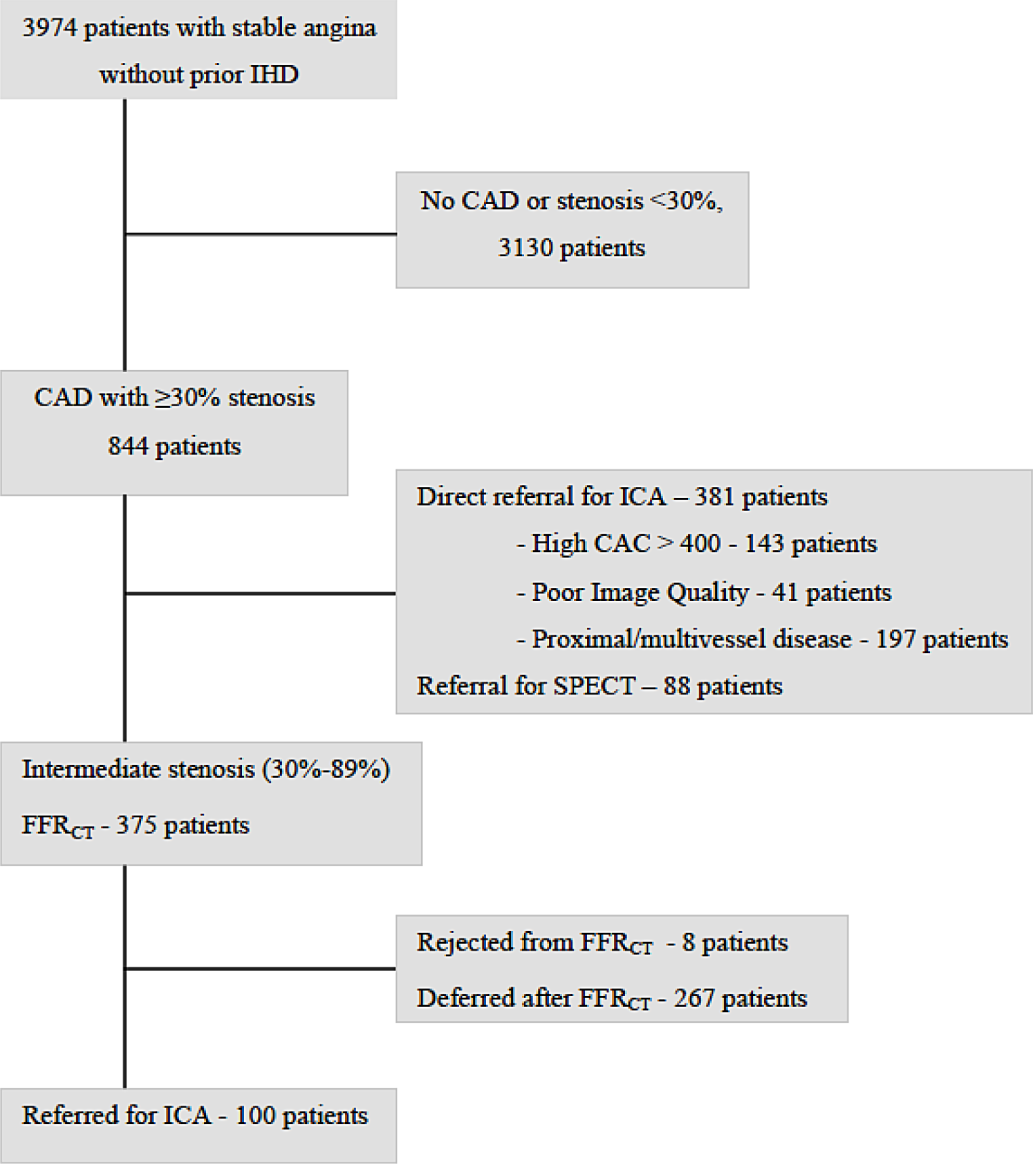
Flow chart. Schematic representation of flowchart for patients with new onset SAP and suspected coronary stenosis, who are eligible for coronary CTA and referral for FFR_CT_. Abnormal FFR_CT_ test: An FFR_CT_ value ≤ 0.80, registered 10–20 mm distal to the stenosis (2 cm-FFR_CT_) was the primary criterium for abnormality. Distal in vessel FFR_CT_, ∆FFR_CT_ (difference of FFR_CT_-values immediately proximal and 10 mm distal to stenosis), high risk plaque features, plaque burden, stenosis location and number of stenosis (21) represented alternative criteria for abnormality. Abnormal MPI-SPECT: Diagnosis of an abnormal test result based on traditional criteria, including a summed difference score ≥ 2/an ungated stress-and-rest volume ratio of > 1.19/a significant decrease in left ventricular ejection fraction from rest to stress. Abbreviations: SAP: stable angina pectoris CTA: computed tomography angiography CAD: coronary artery disease OMT: optimal medical treatment FFR_C_: coronary computed tomography angiography derived fractional flow reserve ICA: invasive coronary angiography MPI-SPECT: myocardial perfusion imaging by single-photon emission computerized tomography SDS: summed difference score

Two-center, retrospective all-comer study. Data represents the initial clinical results following implementation of FFR_CT_ as the preferred test for functional assessment of intermediate stenosis by CTA in patients with new onset suspected SAP, Fig. 1. Data were collected in 2018 or 2019 at two departments of cardiology at University Hospital of Southern Denmark (Vejle and Esbjerg), Region of Southern Denmark. Only patients with sinus rhythm, a body mass index *≤* 40 kg/m^2^, an estimated glomerular filtration rate *≥* 45 ml/min, and no previous revascularization were eligible for CTA. Patients with left main disease, multivessel disease or severe proximal disease by CTA were referred directly to ICA according to best practice guidelines [5, 20]. Clinical data were obtained from electronic patient journals. This study was approved by regional authorities (journal nr.: 21/10587 and 18/44285).

### Coronary CTA

CTA was performed using either a SOMATOM Definition Flash or a FORCE CT scanner (both from Siemens Healthineers, Forchheim, Germany). Oral beta-blockers or ivabradine were administered if necessary, targeting a heart rate ≤ 60 beats/min. All patients received sublingual nitroglycerin. An initial non-enhanced scan for calcium scoring was performed. CTA was assessed by skilled CT cardiologists. Vessels *≥* 2 mm in diameter were evaluated and graded visually by the interpreters. Stenosis severity by CTA was classified as; non-obstructive, 1–29%; suspected obstructive 30–89%; obstructive *≥* 90%. Suspected obstructive stenosis was divided into categories 30–69% and 70–89% stenosis. Information regarding stenosis severity was obtained by reviewing CTA interpretation reports in the electronic patient journal.

### FFR_CT_-analysis and interpretation

Standard acquired coronary CTA datasets were transmitted for central analysis (HeartFlow Inc., Redwood City, California) as previously described. A generated individualized 3D-model of the FFR_CT_-analysis served as the platform for registration and interpretation of the FFR_CT_-data. Interpretation of FFR_CT_-data and decisions on referral to ICA were performed by skilled CT cardiologists and were guided by current recommendations for interpretation of FFR_CT_-data [21]. Briefly, an FFR_CT_ value ≤ 0.80, registered 10–20 mm distal to a stenosis (2 cm-FFR_CT_) was the primary criterion for classifying a stenosis as hemodynamic significant and the patient as potential candidate for ICA. Alternative criteria for referral to invasive procedures included severity of decrease of distal in vessel FFR_CT_, magnitude of ∆FFR_CT_ (difference of FFR_CT_-values immediately proximal and 10 mm distal to stenosis), high risk plaque features (positive remodeling, spotty calcification or low-attenuation plaque), plaque burden, stenosis location, and number of stenoses [21]. 

### Invasive procedures and revascularization

Diagnostic ICA was performed at the two CTA hospitals. A multidisciplinary heart team conference and/or the treating physician made decisions on revascularization strategy. Patients were classified as having obstructive CAD, if ≥ 1 coronary vessel had *≥* 50% stenosis (visual assessment), or if ≥ 1 coronary stenosis had an FFR-value *≤* 0.80 distal to stenosis. Percutaneous coronary intervention (PCI) or coronary artery bypass grafting (CABG) were performed at one tertiary hospital and in accordance with international guidelines [4, 22].

### Statistical methods

Baseline characteristics are presented as mean (SD) or medians (interquartile range [IQR]) as appropriate for continuous variables and proportions for categorical variables. Logistic regression was used to compare coronary CTA versus FFR_CT_ with respect to incidences of obstructive CAD by ICA and revascularization rates, and to assess differences in revascularization rate according to the applied FFR_CT_-interpretation algorithm (2 cm distal to stenosis criterium versus alternative criteria) for stenosis categories 30–69% and 70–89%.

Diagnostic performance of baseline risk variables (diabetes, hypertension, dyslipidaemia, and smoking), coronary stenosis at CTA, symptoms, and coronary CTA–derived FFR were assessed using receiver operating characteristic curves, and differences between areas under the receiver operating characteristic curve were evaluated using the DeLong method.

A p-value of < 0.05 was considered statistically significant. All statistical analyses were performed using Stata version 16.1 software (Stata Corp, College Station, Texas).

## Results

Coronary CTA was performed in 3974 patients: 3130 (79%) had no CAD or stenosis < 30%, 381 (10%) were referred directly to ICA, and 463 (12%) were referred for non-invasive functional assessment, Fig. 1. In patients undergoing non-invasive functional testing, single photon emission computerized tomography (SPECT) myocardial perfusion imaging was performed in 88 (19%) patients and FFR_CT_ in 375 (81%). FFR_CT_ analysis was successful in 367 (98%) patients and 8 (2%) were rejected based on poor image quality due to coronary calcification, misalignment and/or motion artifacts. Turn-around time for FFR_CT_ was < 48 h in all patients. Baseline characteristics of patients referred to ICA, directly after CTA or after selective FFR_CT_ are shown in Table 1.

**Table 1:** Baseline characteristics of patients referred to invasive coronary angiography according to non-invasive testing strategy

	CTA-guided *n* = 381	FFR_CT_-guided *n* = 367	*p*-value
**Demographics**			
Age	66 ± 10	64 ± 10	0.007
Gender, male	244 (64)	228 (61)	0.621
**Risk factors**			
Diabetes	53 (14)	40 (11)	0.213
Hypertension	262 (69)	218 (58)	0.008
Dyslipidemia	259 (68)	236 (63)	0.289
Current smoker	268 (70)	209 (56)	< 0.001
**Coronary CTA**			
Agatston score, U	574 (198–1234) [0-6067]	211 (81–456) [0-3168]	< 0.001
Stenosis severity			
30–69%	38 (10)	319 (87)	< 0.001
70–89%	84 (22)	48 (13)	
*≥*90%	75 (20)	0 (0)	
Non evaluable*	184 (48)	0 (0)	

Values given as n (%), mean ± SD or median (interquartile range) [range]

*Due to high calcium (*n* = 143) or poor image quality (*n* = 41)

Patients classified according to most severe stenosis

Abbreviations: CTA = computerized tomography angiography; FFR_CT_=coronary CTA derived fractional flow reserve; ICA = invasive coronary angiography

**Fig. 2:**
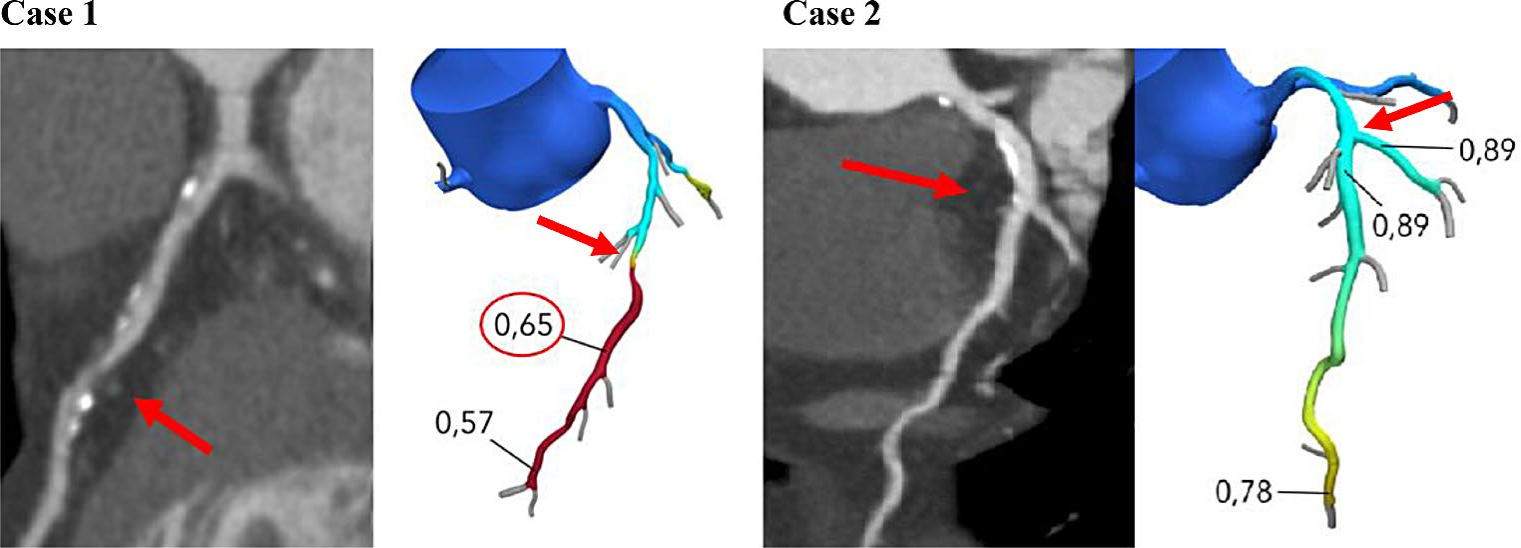
Case examples. Representation of two patient cases with proximal (70–89%) LAD stenosis. Case 1 was referred to ICA and was revascularized and treated with OMT. Case 2 was deferred from further testing and treated with OMT Arrows indicate location of stenoses. Markers illustrate the point in the coronary tree 2 cm distal to stenoses, where FFR_CT_ values were registered *Abbreviations* LAD = left anterior descending artery; ICA = invasive coronary angiography; OMT = optimal medical therapy; FFR_CT_=coronary computed tomography angiography derived fractional flow reserve

The clinical consequences of using selective FFR_CT_ testing for decision making are shown in Table 2, case examples in Fig. 2. FFR_CT_ had a positive predictive value of 62%, and 71% of patients referred for FFR_CT_ were deferred from ICA or other downstream tests, Table 2. The proportion of patients who were deferred from ICA based on the FFR_CT_ test result was 80% (*n* = 255) in those with a 30–69% stenosis and 25% (*n* = 12) in those with a 70–89% stenosis.

**Table 2:** Downstream testing and treatment after selective FFR_CT_ testing in stable angina pectoris

No need for additional downstream testing		267 (71)
Referred for ICA		100 (27)
Rejected FFR_CT_ analysis		8 (2)
**Findings by ICA and treatment**		
Obstructive CAD		62 (62)
*Revascularization*	53 (53)	
1-vessel PCI	43 (81)	
2-vessel PCI	6 (11)	
3-vessel PCI	0 (0)	
CABG	4 (8)	
*OMT*	9 (9)	
Non-obstructive CAD, OMT		38 (38)
Turn-around time < 48 h		367 (98)

Values given as n (%) Obstructive CAD, n (%) was defined by eye-balling by the interventionist, 49 (79) or by a measured fractional flow reserve *≤* 0.80, 13 (21). Non-obstructive CAD was defined by eye-balling in 34 (89%) or by invasive FFR *≤* 0.80 in 4 (11%). *Abbreviations* CABG = coronary arterial bypass grafting; CAD = coronary artery disease; CTA = computerized tomography angiography; FFR_CT_=coronary CTA derived fractional flow reserve; ICA = invasive coronary angiography; OMT = optimal medical therapy; PCI = percutaneous coronary intervention

**Fig. 3:**
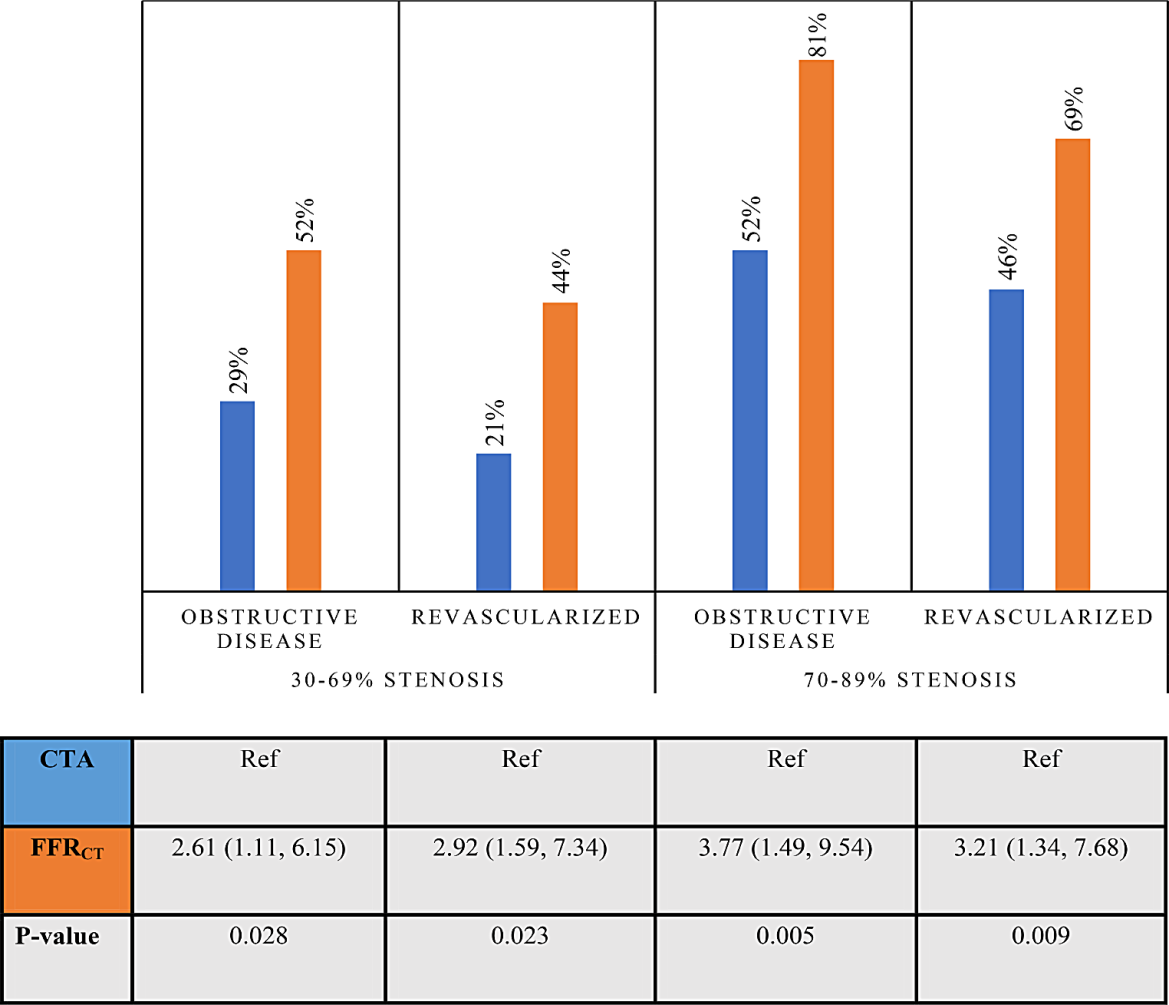
FFR_CT_-guided versus coronary CTA-guided referral to ICA in suspected coronary stenosis. Invasive findings and treatment. The diagram illustrates findings by ICA and treatment depending on coronary CTA-guided or FFR_CT_-guided referral to ICA. The number of patients, n, referred directly to ICA according to stenosis severity by coronary CTA were: 30–69%, 38; 70–89%, 84. The corresponding numbers for patients referred based on FFR_CT_-testing were: 30–69%, 64; 70–89%, 36. Differences in findings by ICA/revascularization rates between referral practices are given as odds ratios (95% confidence intervals). *Abbreviations* ICA = invasive coronary angiography; coronary CTA = coronary computerized tomography angiography; FFR_CT_=coronary CTA derived fractional flow reserve

The finding that FFR_CT_ superiorly detected obstructive disease and carried a higher revascularization rate compared with CTA alone was observed both in patients with a 30–69% stenosis and in those with a 70–89% stenosis, Fig. 3. Patients referred to ICA based on CTA without FFR_CT_ were older, had more risk factors for cardiovascular disease, a higher coronary artery calcium score, and a more severe degree of CTA-assessed stenosis when compared to patients referred to ICA after FFR_CT_, Table 1. Invasive FFR was performed in 11% of patients in the CTA group and in 21% of patients in the FFR_CT_ group.

Of the 100 patients referred to ICA after FFR_CT_, 85 patients met the primary criterium of a 2 cm-FFR_CT_ value ≤ 0.80, while 15 patients had the alternative criteria for referral to ICA. Revascularization rates were: primary criterium, 51 (60%), versus alternative criteria, 2 (13%), OR (95% CI) 9.75 (2.07, 45.97) p-value < 0.005. In 100 patients referred to ICA by FFR_CT_, revascularization was guided by visual assessment in 83 (83%) and by FFR in 17 (17%). There was no difference in revascularization rates between patients in whom treatment decisions were based on visual assessment, 49 (59%), compared to FFR, 13 (76%), *p* = 0.185.

Amongst the 15 patients referred to ICA based on the alternative criteria, the main drivers were a high delta FFR_CT_ (*n* = 13), and/or a distal FFR_CT_ <0.80 (*n* = 12), and/or a proximal stenosis (*n* = 11), and/or multivessel stenoses (*n* = 6).

In total, 184 patients were referred directly to ICA from CTA without using FFR_CT_ as second line test. When this was due to high content of coronary calcification (*n* = 143, 78%), obstructive CAD by ICA was present in 69 patients (48%) and revascularization was performed in 54 (38%). When FFR_CT_ was not performed due to image quality, obstructive CAD by ICA was present in 10 (24%) and revascularization was performed in 7 (17%).

**Fig. 4:**
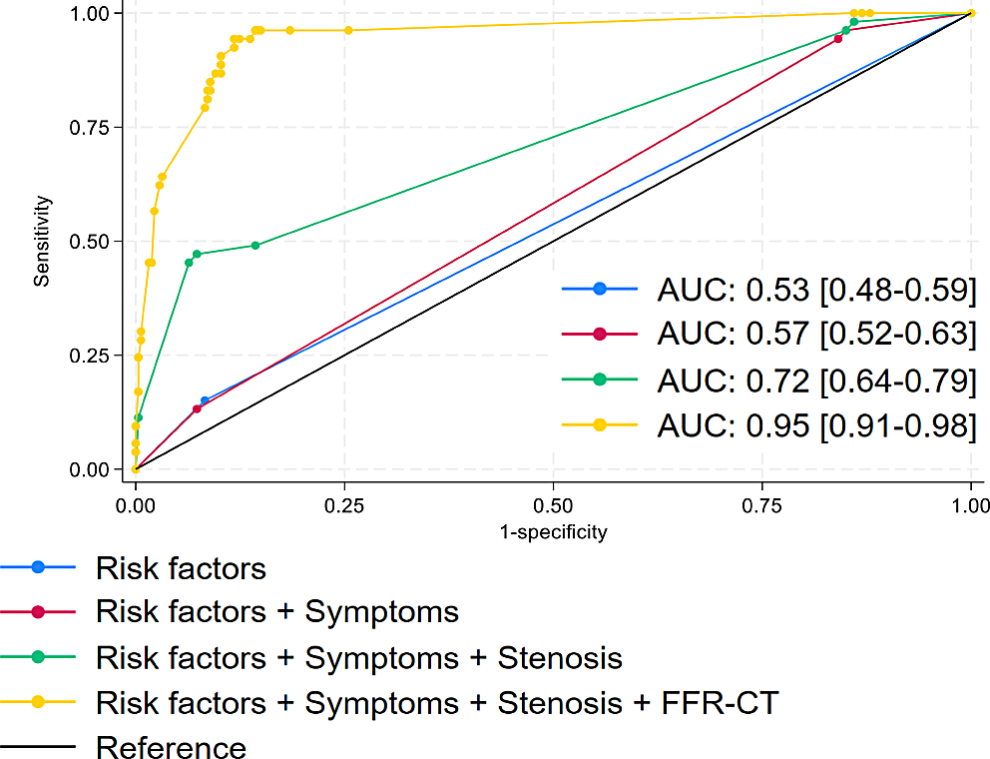
Graphs show performance evaluation of models created using combinations of baseline participants characteristics for discriminating the prediction of revascularization. Receiver operating characteristics performed best for discriminating when adding FFR_CT_ to risk factors (diabetes, hypertension, dyslipidemia, and smoking), symptoms (dyspnoea and chest pain) and stenosis (+/-70%), AUC 0.95 [0.91–0.98], risk factors alone with AUC 0.53 [0.48–0.59], Risk factors and symptoms, AUC 0.72 [0.64–0.79] and risk factors, symptoms and stenosis AUC 0.72[0.64–0.79], *p* < 0.001

Receiver operating characteristics curve analysis showed that the addition of FFR_CT_ to baseline risk variables (diabetes, hypertension, dyslipidemia, and smoking), symptoms and degree of stenosis improved overall discrimination to the prediction of revascularization AUC 0.95[0.91–0.98] vs. AUC 0.72 [0.64–0.79] (*P* < 0.001), Fig. 4.

Amongst the 53 patients that were revascularized in the FFR_CT_ group, FFR_CT_ correctly detected the culprit vessel in 51 cases (93%), whereas another vessel was revascularized in 2 cases (7%).

## Discussion

In this study of stable angina patients examined with coronary CTA at referral hospitals without PCI facilities, we found FFR_CT_ to be a feasible second line test that reduced the need for invasive procedures and increased the revascularization rate as compared with coronary CTA alone.

The high proportion of patients that were deferred from downstream diagnostic testing following a normal FFR_CT_ analysis in the present study is in line with previous studies, in which the safety of using an FFR_CT_ based approach for guiding deferral from ICA was documented [12–14, 18, 23, 24]. In particular, our results are in line with the ADVANCED multicentre registry [18], in which 2/3 of all patients referred for FFR_CT_ were deferred from invasive procedures and further tests. The observed higher revascularization rates associated with an FFR_CT_-guided approach as compared to decision-making based on visual assessment by experienced CT-cardiologists corresponds with previous head-to-head comparisons, showing a better diagnostic performance when adding FFR_CT_ to conventional CTA for prediction of the hemodynamic significance of intermediate stenosis [3, 8, 9]. In our study, the benefit of FFR_CT_ was observed despite the fact that patients referred to ICA without FFR_CT_ had more risk factors, more central lesions, and a higher degree of stenosis by CTA than patients in the FFR_CT_ group. The ROC analysis supports that FFR_CT_ improved the ability to predict obstructive coronary disease in our study population.

Our post hoc analysis provides an opportunity to evaluate some aspects of the initial clinical experiences of implementing FFR_CT_ in an all-comer population. We observed a higher revascularization rate if referral to ICA was based on the recommendations [20] for interpretation of FFR_CT_, applying lesion-specific criteria, as compared to referral driven by alternative criteria for FFR_CT_ test abnormality. However, some studies have shown alternative criteria like the delta FFR_CT_ value to be an important predictor of revascularization. [25]. We also report that revascularization in some cases was deferred by PCI operators despite a positive FFR_CT_. This was not only due to false positive FFR_CT_ results as compared to invasive measurements, but also based on an overall clinical judgment including comorbidity, burden of symptoms, and localization and extent of calcification of stenosis. Possibly, such aspects are more likely to be observed in a real world setting than in protocolled trials.

In our study, 19% of patients were referred for myocardial perfusion imaging (MPI) mainly because the quality of the CT-scan was not suitable for FFR_CT_, a strategy that is recommended according to contemporary guidelines [4, 5]. The results of downstream testing in this group of patients were not registered in this study. However, we have previously reported that SPECT as a second-line test strategy following CTA had a sensitivity of 41% and a specificity of 86% as compared to invasive FFR [10], which is similar to the results obtained in the Dan-NICAD trial [26]. Thus, FFR_CT_ has been associated with a better over all diagnostic performance and a higher sensitivity than perfusion imaging with SPECT and magnetic resonance [10, 11].

Overall, FFR_CT_ testing seems well-suited for implementation in the diagnostic algorithm for patients with suspected SAP. First, patients do not need to physically attend additional examinations, as the FFR_CT_ analysis is generated from available coronary CTA data sets, thus minimizing patient discomfort. Second, overall exposure to radiation and contrast is reduced. Third, the FFR_CT_ test result is available within 24 h after CTA, whereas referring patients to other second-line tests after CTA would generally carry a greater delay of the diagnostic process. Fourth, FFR_CT_ has demonstrated excellent diagnostic performance, also in patients with a high CAC [15, 27, 28] or aortic stenosis [29, 30], and a normal test result is associated with a good prognosis [12–15]. Fifth, recent studies [31, 32] have indicated that implementation of FFR_CT_ is cost neutral or cost effective as compared to traditional testing strategies. One study indicated, that CTA/FFR_CT_ may be the most cost-effective strategy in patients with stenoses > 50% [33]. In addition, FFR_CT_ has proven useful in guiding individual antianginal therapy, whether the treatment is optimal medical treatment or PCI [34, 35].

The present study comprises real-world data obtained from two referral hospitals without PCI-facilities. We found FFR_CT_ to be an effective tool that minimized the number of invasive procedures and increased the revascularization rate.

## Limitations

It should be emphasized that this was a retrospective study of patients included at 2 hospitals. As the study is based on thousands of consecutive patients undergoing CTA, it might be claimed that the number of patients with an intermediate stenosis suitable for FFR_CT_ analysis was rather low.

Slightly different patient approaches in terms of utilization of MPI-SPECT/FFR_CT_ and referral practice to ICA following CTA and the fact that management and revascularization decisions were at the discretion of the treating physicians make the results subject to potential selection bias. In the real world setting of our study, we found that invasive FFR was performed in a relatively small proportion of patients and it may be speculated that a positive FFR_CT_ result influenced operators not to perform FFR before revascularization. A few patients were referred directly from CTA to ICA as the CT-image quality was considered inadequate for FFR_CT_-analysis. The low revascularization rate (17%) amongst these patients indicates that repeat CTA to optimize image quality should probably have been performed instead of direct referral to ICA. At the time of this study, patients with CAC > 400 were sometimes referred directly to ICA and sometimes to further functional testing, which constitutes a limitation.

It should also be mentioned that only one CT-vendor was applied for performing CTA and only one company providing FFR_CT_ was used.

## Conclusion

Implementation of FFR_CT_ at referral hospitals without PCI-facilities is feasible, implies minimal need for alternative downstream non-invasive testing, leads to a substantial reduction of invasive procedures and increases the revascularization rate in patients with recent onset stable angina pectoris and intermediate stenosis by CTA.

## Data Availability

The data that underlines this article is available in the manuscript.
